# Brain Calcifications Secondary to Idiopathic Hyperthyroidism and Hypoparathyroidism

**DOI:** 10.31486/toj.23.0004

**Published:** 2024

**Authors:** Bushra Zafar Sayeed, Faiza Zafar Sayeed, Muhammad Nashit, Shaheen Bhatty

**Affiliations:** ^1^ Department of Medicine, Dr. Ruth K. M. Pfau Civil Hospital Karachi, Karachi, Pakistan

**Keywords:** *Calcification–physiologic*, *endocrinology*, *hyperthyroidism*, *hypoparathyroidism*

## Abstract

**Background:** Thyroid and parathyroid hormones are essential components of the metabolic system and its regulation. Concurrent hyperthyroidism with hypoparathyroidism is an extremely rare finding and is not considered a common etiology of brain calcifications seen on imaging. Brain calcifications can cause a range of neurologic symptoms, including movement disorders, cognitive impairment, and seizures. Prompt recognition and treatment of hypoparathyroidism are essential to prevent or minimize the development of brain calcifications and associated neurologic symptoms.

**Case Report:** A 39-year-old female presented to the emergency department in an unconscious state with generalized weakness and tonic-clonic seizures for 1 day. On clinical examination, she had jerky movements of her upper limbs, and her Glasgow Coma Scale score was 4/15. Supporting hypoparathyroidism, she had low levels of serum parathyroid hormone, calcium, and vitamin D and a high level of serum phosphorus. Her magnesium level was normal. Thyroid profile revealed hyperthyroidism. Noncontrast-enhanced computed tomography scan at the midbrain level showed multiple bilateral hyperintense areas in the basal ganglia and thalami suggestive of calcifications. The patient was treated with calcium and vitamin D supplements and antithyroid agents that successfully resolved her symptoms.

**Conclusion:** This case provides important documentation for including hypocalcemia as a result of hypoparathyroidism in the differential diagnosis of patients with seizures. The treatment approach used with our patient can be considered for managing seizures in cases where the underlying cause is challenging to identify. This case highlights the importance of a thorough evaluation and individualized treatment plan for patients with seizures.

## INTRODUCTION

Thyroid and parathyroid hormones are essential components of the metabolic system and its regulation. Hyperthyroidism primarily manifests with features of an increased basal metabolic rate: tremors, palpitations, anxiety, weight loss, increased appetite, heat intolerance, diarrhea, shortness of breath, and menstrual irregularities. The most common cause of hyperthyroidism is Graves disease. Other causes include toxic multinodular goiter, thyroiditis, iodine-induced thyroid dysfunction, and exogenous increased thyroid hormone intake.^1^

On the other hand, hypoparathyroidism manifests with features of decreased extracellular calcium levels and can result in perioral tingling and numbness, muscle cramps, tetany, parkinsonism, and neuromuscular dysfunction. Calcium deposition in organs such as the kidney and brain is common. The most common cause of hypoparathyroidism is neck surgery that causes damage to the parathyroid gland or removes it. Almost 75% of cases have this etiology.^2^ Parathyroid hormone (PTH) maintains homeostasis of vitamin D and phosphorus levels through its effects on the kidney. PTH activates the renal 1-α hydroxylase enzyme, which leads to activation of 25-hydroxy vitamin D and also inhibits renal tubular phosphate reabsorption. The deficiency of this hormone thus leads to low serum vitamin D levels and high serum phosphorus.^3^ The initial investigation of hypoparathyroidism should also include serum magnesium level, as both high and low levels may cause functional hypoparathyroidism.^4^

We report the case of a female with generalized tonic-clonic seizures who was diagnosed with brain calcifications secondary to hypocalcemia and hyperphosphatemia caused by idiopathic hypoparathyroidism concurrent with hyperthyroidism.

## CASE REPORT

A 39-year-old female with no known comorbidities presented to the emergency department in an unconscious state with generalized weakness and generalized tonic-clonic seizures for 1 day. She had a history of gradually progressive generalized weakness, behavioral disturbance, body aches, palpitations, irritability, and jerky movements of the extremities for the prior 5 months. Family members reported that she had become increasingly irritable and suspicious of family members and started having mood swings within the prior 5 months. The patient had no history of surgery and was taking no routine medications.

**Figure 1.**
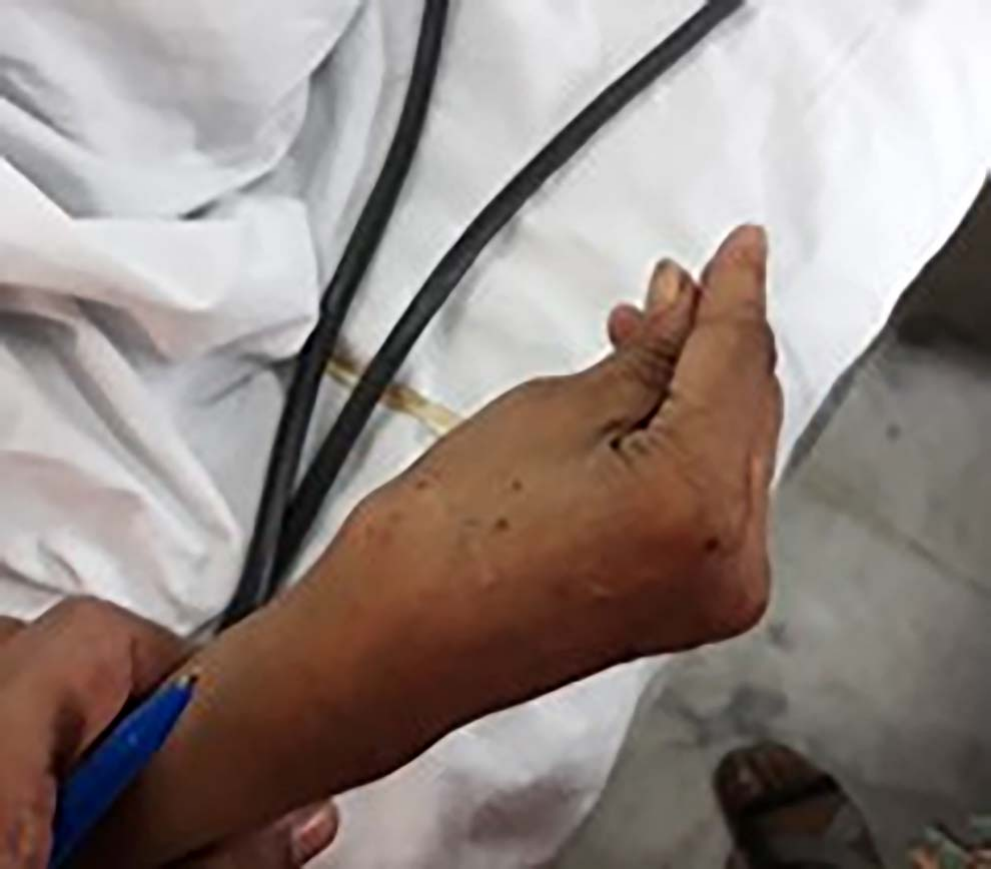
Trousseau sign in the right hand.

On clinical examination, the patient had jerky movements of her upper limbs, tingling, and numbness in her hands and feet. She had positive doll's eyes reflex, neck rigidity, and Trousseau sign (Figure 1) in the right hand. Her Glasgow Coma Scale score was 4/15. No enlargement of the thyroid gland was noticed.

Laboratory investigations (Table) revealed low serum calcium, serum vitamin D, serum thyroid stimulating hormone (TSH), and PTH; high serum phosphorus; and normal magnesium and albumin levels.

Levels for inflammatory markers—creatine phosphokinase, erythrocyte sedimentation rate, and C-reactive protein—were increased, and the patient's thyroid profile revealed hyperthyroidism. Autoimmune tests were negative, ruling out autoimmunity as a cause of hyperthyroidism and hypoparathyroidism. Osteomalacia and renal osteodystrophy were ruled out using dual-energy x-ray absorptiometry scan and plain film x-ray, respectively. Pseudohypoparathyroidism was excluded by the absence of brachydactyly and dwarfing.

**Table.** Patient's Laboratory Values at Admission

Test	Value	Reference Range
**Complete blood count**		
Hemoglobin, g/dL	10.3	12.1-15.1 (females)
Hematocrit, %	32	36-48 (females)
Mean corpuscular volume, fl	86	80-100
White blood cells, μL	10,500	4,000-11,000
Platelets, μL	387,000	150,000-450,000
**Renal function tests**		
Blood urea nitrogen, mg/dL	25	6-24
Creatinine, mg/dL	1.3	0.6-1.1 (females)
Sodium, mEq/L	141	135-145
Potassium, mEq/L	3.6	3.6-5.2
Chloride, mEq/L	98	96-106
Bicarbonate, mEq/L	22.3	22-29
**Calcium, vitamin D, and parathyroid hormone**		
Calcium, mg/dL	5.2	8.5-10.2
Corrected calcium, mg/dL	5.8	
Phosphorus, mg/dL	7	2.8-4.5
Magnesium, mg/dL	2.2	1.7-2.2
Vitamin D, ng/mL	17	30-50
Parathyroid hormone, pg/mL	2.08	10-55
**Thyroid profile**		
Thyroid stimulating hormone, μIU/mL	<0.1	0.4-4.5
Free T3, pmol/L	9.1	2.0-7.0
Free T4, ng/dL	3.6	0.9-2.3
**Thrombophilia profile**		
Prothrombin time, sec	10	11-13.5
Activated partial thromboplastin time, sec	26	21-35
International normalized ratio	0.9	0.8-1.1
**Liver function tests**		
Total bilirubin, mg/dL	0.5	0.1-1.2
Alanine transaminase, IU/L	42	7-55
Aspartate aminotransferase, IU/L	25	8-48
Alkaline phosphatase, IU/L	210	44-147
Gamma-glutamyl transpeptidase, IU/L	48	0-30
**Viral markers**		
Hepatitis B surface antigen	Negative	
Hepatitis C antibody	Negative	
**Autoimmune profile**		
Antinuclear antibodies	Negative	
Anti-double stranded DNA	Negative	
Antimitochondrial antibodies	Negative	
Anti-TPO	Negative	
Antithyroglobulin antibody	Negative	
**Nutritional profile**		
Iron, μg/dL	72	50-150
Ferritin, μg/L	350	24-336
Total iron binding capacity, μg/dL	270	250-450
Vitamin B12, ng/L	587	200-1,100
Folate, ng/mL	10	6-40
**Other tests**		
Total protein, g/dL	6.0	6.0-8.3
Albumin, g/dL	3.5	3.4-5.4
Globulin, g/dL	2.5	2.0-3.9
Albumin/globulin ratio	1.5	1.1-2.5
Erythrocyte sedimentation rate, mm/h	124	0-29 (females)
C-reactive protein, mg/L	54	8-10
Creatine phosphokinase, U/L	1,480	30-135 (females)

**Figure 2.**
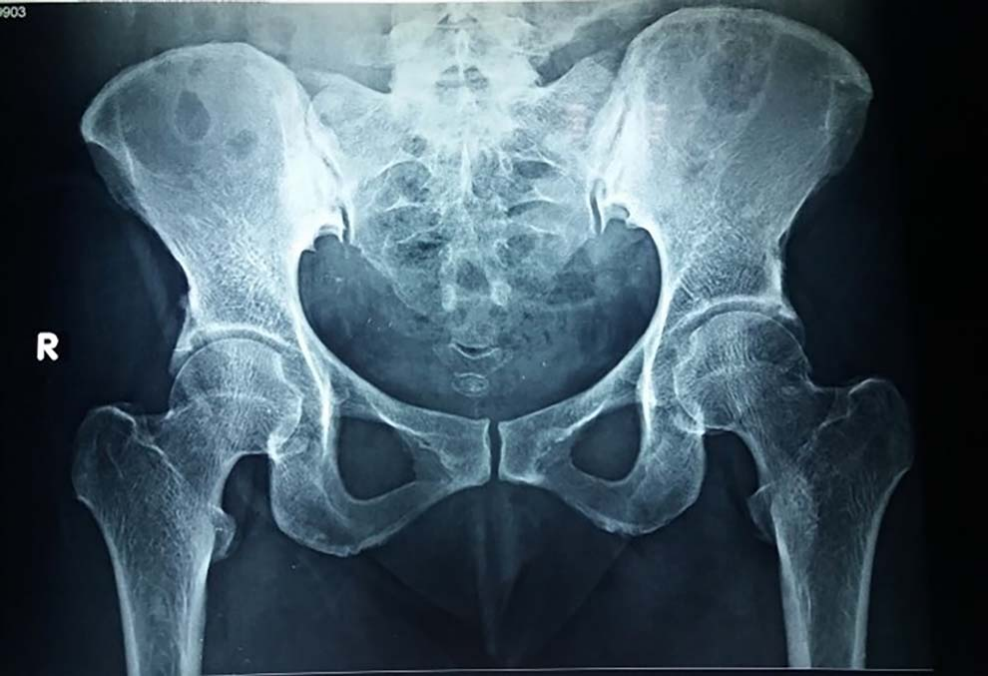
Pelvic x-ray in anteroposterior view shows ossification of the iliolumbar, sacrospinous, and sacrotuberous ligaments and the acetabular margins.

Electrocardiogram showed QTc prolongation of 516 msec and T-wave inversions in leads V1, V2, and V3, supporting hypoparathyroidism. Pelvis radiography showed ossification of iliolumbar, sacrospinous, and sacrotuberous ligaments and the acetabular margins (Figure 2).

**Figure 3.**
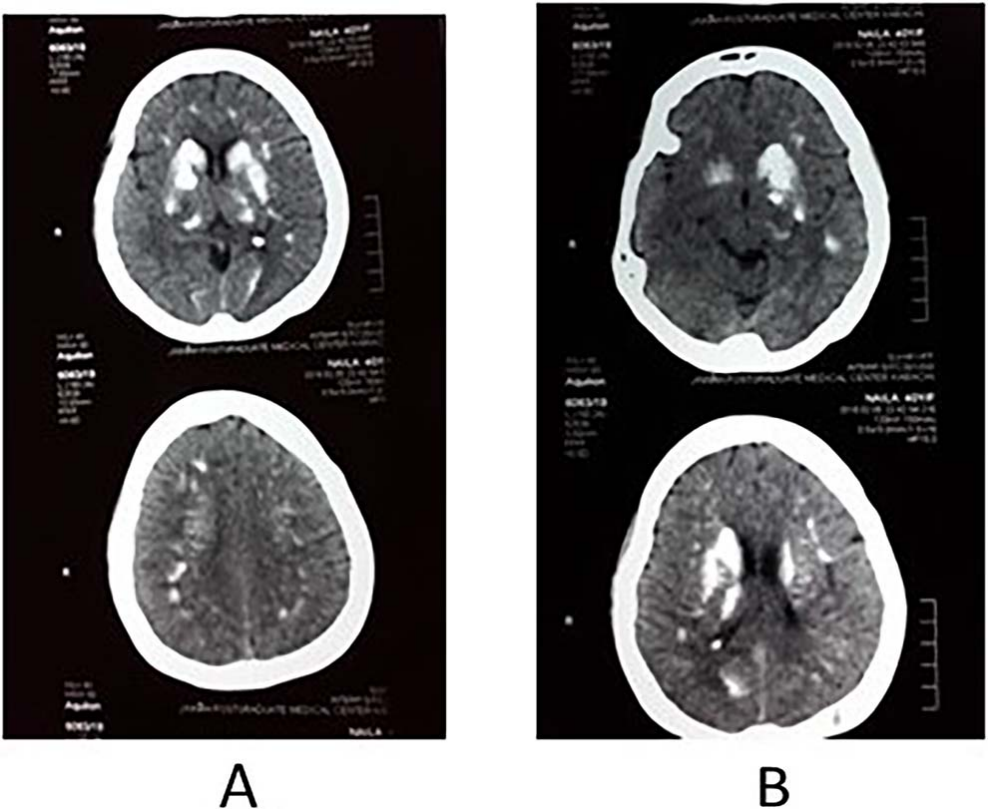
Noncontrast-enhanced computed tomography scan of the (A) left and (B) right cerebral hemispheres showing calcifications in the basal ganglia and thalami.

Noncontrast-enhanced computed tomography (CT) scan at the midbrain level showed multiple bilateral areas of calcification in the basal ganglia and thalami due to hypoparathyroidism (Figure 3).

The thyroid scan confirmed diffusely increased uptake of technetium-99m pertechnetate bilaterally. Idiopathic hyperthyroidism and hypoparathyroidism were diagnosed.

During a hospital stay of 1 week, the patient's calcium level was increased by administration of intravenous calcium gluconate. After clinical improvement and a reduction in symptoms, the patient was switched to an oral calcium tablet (Qalsan D, containing 500 mg elemental calcium and 125 IU of vitamin D3) taken 3 times daily and 3 tablets of Neo-Mercazole (carbimazole) 5 mg taken 3 times daily for 3 months during which TSH was monitored at 6-week intervals. This treatment regimen resulted in complete resolution of the patient's symptoms 2 months postdischarge as shown by calcium and TSH levels of 9.2 mg/dL and 3.1 μIU/L, respectively. The patient was followed monthly for 3 months, and she is currently on 3-month follow-up in the departments of internal medicine and endocrinology.

## DISCUSSION

Only 3 cases of idiopathic hypoparathyroidism with concomitant hyperthyroidism have been reported.^5-7^ Dahl et al reported the first case in 1962.^5^

Physical symptoms and laboratory investigations are vital to diagnosing concomitant idiopathic hypoparathyroidism with hyperthyroidism and devising a management plan. Idiopathic hypoparathyroidism with concurrent hyperthyroidism typically presents with symptoms of seizures, psychosis, and basal ganglia calcification. Idiopathic hypoparathyroidism-induced seizures have been mistreated as epilepsy.^8^ Therefore, an essential part of the investigation process at the initial presentation is the exclusion of any electrolyte abnormalities, primarily sodium, calcium, and magnesium abnormalities.^8^ Ning et al described a case of idiopathic hypoparathyroidism with hypocalcemia and intracranial calcification.^9^ The patient's presenting symptoms included depression, anxiety, agitation, and sweating. After correction of her calcium level, the patient's symptoms markedly improved.^9^

Seizures may be a presenting sign of hypocalcemia preceding tetany and chorea. Twenty percent to 25% of patients with acute hypocalcemia and 30% to 70% of patients with idiopathic hypoparathyroidism have shown the presence of seizure activity.^10^

The calcifications in the basal ganglia and thalami of our patient were a manifestation of idiopathic hypoparathyroidism concurrent with hyperthyroidism. Eaton et al first documented basal ganglia calcification in 1939.^11^ Mejdoubi and Zegermann described a 24-year-old patient with symmetrical bilateral calcifications in the basal ganglia, cerebellum, and at the grey-white junction secondary to hypoparathyroidism.^12^ While hypoparathyroidism is one of the most common causes of intracranial calcifications, the differentials include Fahr disease, carbon monoxide poisoning, central nervous system tuberculosis, TORCH (toxoplasmosis, others [syphilis, hepatitis B], rubella, cytomegalovirus, and herpes simplex) infections, Cockayne syndrome, and mitochondrial disease.^12,13^

Rizvi et al described generalized seizures with extrapyramidal features of tremors and rigidity in a patient with hypoparathyroidism.^14^ Laboratory investigations revealed hypoparathyroidism, and a head CT scan showed calcification of the subcortical white matter of the frontal and parietal lobes, cerebellum, and bilateral basal ganglia. The patient recovered after normal serum calcium and TSH levels were restored with oral medications.

Two classic indicators of hypocalcemia are Trousseau sign and Chvostek sign. Our patient demonstrated a positive Trousseau sign. Additionally, she was extremely irritable, with frequent mood swings and episodes of paranoia, and was becoming increasingly aggressive toward and suspicious of family members. These findings are consistent with the psychosis associated with the hormonal imbalance in hypoparathyroidism. Hyperthyroidism is also associated with mood disturbances, as illustrated in a case by Marian et al^15^ in which the patient presented with depressive symptoms and later developed delirious hallucinatory symptoms. The physical symptoms of sweating, palpitations, and tremors and the examination findings of goiter and exophthalmos are suggestive of hyperthyroidism.^15^

Our patient's electrocardiogram showed a prolonged QT interval, and Davis et al have proposed that hypocalcemia is the cause of this prolongation.^16^ The most likely mechanism is the increased phase 2 of the cardiac muscle action potential, which increases the ST segment and the QT interval.

Long-standing hypoparathyroidism can also cause spondyloarthropathy.^17^ Pelvic radiography of our patient showed ossification of iliolumbar, sacrospinous, and sacrotuberous ligaments and the acetabular margins. Similar features are seen in conditions such as ankylosing spondylitis and diffuse idiopathic skeletal hyperostosis.^18^ However, no erosive arthropathy or features of sacroiliitis are observed in patients with hypoparathyroidism. Enthesopathy, primarily at the lesser and greater trochanters, ischial tuberosities, and iliac crests, is a complication of hypoparathyroidism.^19^

Acquired and hereditary causes of hypoparathyroidism were ruled out for our patient, based on the family history, autoimmune profile, and surgical history. However, whether idiopathic hypoparathyroidism is an autoimmune process is debatable, as antibodies have been detected in patients.^20^

Because of the absence of surgery or radioiodine therapy in our patient's history, the most likely cause of her low PTH level was presumed to be idiopathic hypoparathyroidism, and the cause of her hyperthyroidism may have also been idiopathic given the absence of autoantibodies to the thyroid gland.

The treatment plan for the patient consisted of correcting her serum calcium and vitamin D levels. The primary therapies for individuals with hypoparathyroidism are calcium supplements and activated vitamin D, except in individuals whose condition is caused by hypo- or hypermagnesemia. Our patient was also treated with antithyroid drugs for hyperthyroidism.

## CONCLUSION

To our knowledge, only 3 cases of idiopathic hypoparathyroidism with concomitant hyperthyroidism have been previously reported. Idiopathic hypoparathyroidism may escape detection for years because of the lack of characteristic symptoms. As illustrated by our case, the condition may present with neurologic symptoms suggesting several disorders, such as brain tumor or epilepsy, mental disturbances with personality changes, dementia, and psychosis. We recommend a thorough clinical examination and investigation to exclude hormonal or electrolyte imbalance as causes for patients with a significant history of seizures.

## Notes

*This article meets the Accreditation Council for Graduate Medical Education and the American Board of Medical Specialties Maintenance of Certification competencies for Patient Care and Medical Knowledge.*
